# Malaria vectors in Angola: distribution of species and molecular forms of the *Anopheles gambiae *complex, their pyrethroid insecticide knockdown resistance (kdr) status and *Plasmodium falciparum *sporozoite rates

**DOI:** 10.1186/1475-2875-5-2

**Published:** 2006-01-18

**Authors:** Nelson Cuamba, Kwang Shik Choi, Harold Townson

**Affiliations:** 1 Liverpool School of Tropical Medicine, Pembroke Place, L3 5QA, Liverpool, UK; 2 Ministério da Saúde, Instituto Nacional de Saúde, C.P 264, Maputo, Mozambique

## Abstract

**Background:**

Malaria is by far the greatest cause of morbidity and mortality in Angola, being responsible for 50% of all outpatient attendance and around 22% of all hospital deaths, yet by 2003 only 2% of under-5s used insecticide-treated nets. Entomological studies are an essential foundation for rational malaria control using insecticide-treated nets and indoor residual spraying, but there have been no published studies of malaria vectors in Angola over the 27 years of the civil war, to its end in 2002. This paper describes studies arising from a WHO-sponsored visit in support of the National Malaria Control Programme.

**Methods:**

During April 2001, mosquitoes were sampled by indoor pyrethrum spray collection from four sites in the semi-arid coastal provinces of Luanda and Benguela and two sites in Huambo province, in the humid tropical highlands. Collections took place towards the end of the rainy season and were used to determine the *Anopheles *species present, their sporozoite rates and the frequency of a *kdr *allele conferring resistance to pyrethroid insecticides.

**Results:**

A PCR test for the *Anopheles gambiae *complex showed a preponderance of *An. gambiae*, with indoor resting densities ranging from 0.9 to 23.5 per house. Of 403 *An. gambiae *identified to molecular form, 93.5% were M-form and 6.5% S-form. M and S were sympatric at 4 sites but no M/S hybrids were detected. The highest proportion of S-form (20%) was in samples from Huambo, in the humid highlands. *Anopheles funestus *was found at one site near Luanda. The sporozoite rate of mosquitoes, determined by an ELISA test, was 1.9% for *An. gambiae *(n = 580) and 0.7% for *An. funestus *(n = 140). Of 218 *An. gambiae *(195 M-form and 23 S-form) genotyped for the West African *kdr-*resistance allele, all were homozygous susceptible.

**Conclusion:**

*An. gambiae *M-form is the most important and widespread malaria vector in the areas studied but more extensive studies of malaria vectors are required to support the malaria control programme in Angola. These should include standard insecticide resistance biossays and molecular assays that can detect both metabolic resistance and target site insensitivity.

## Introduction

In West Africa, *Anopheles gambiae, Anopheles arabiensis *and *Anopheles funestus *are the main vectors of malaria, although *Anopheles melas*, a member of the *An. gambiae *complex, is known to be a malaria vector in some coastal areas [1-3].

It has been shown that *An. gambiae *comprises two molecular forms, M and S, recognisable from rDNA sequence differences, either in the intergenic spacer [4] or in the internal transcribed spacer [5,6]. The genetic characteristics of these forms and their known geographical distribution have recently been reviewed [6]. Whilst there is considerable restriction to gene flow between these forms, reproductive isolation is not complete, with the result that the knock-down resistance allele (*kdr*), which confers resistance to pyrethroid insecticides and which is assumed to have first developed in the S-form, has subsequently passed from the S-form to the M-form, presumably due to hybrid formation and consequent introgression [7,8]. Recent studies suggest that the resistance kdr allele is now spreading in the M-form in West Africa [9]. The S-form is known to be the most common and widespread in sub-Saharan Africa, and is the only form found in eastern Africa. The distribution of the forms is reasonably well studied in West Africa from Senegal to Cameroon, but less is known of their distribution further south in western Africa.

Malaria is endemic throughout much of Angola territory, and is by far the highest cause of morbidity and mortality. During the period from 1999 to 2002 (last year for which data are available) there were 1.47 million cases of malaria annually, out of a mid-period population of c.11.4 million. Malaria continues to be responsible for 50% of all outpatient attendance and around 25% of all hospital deaths, yet by 2003 only 2% of under-5s used insecticide-treated nets [10]. Due to the successive wars, malaria vector control activities and operational studies have been interrupted for decades, with a consequent lack of basic information on malaria vectors. This lack of information plus the dearth of skilled malaria entomologists are potential impediments to the goal of the National Malaria Control Programme of scaling up the use of insecticide-treated nets (ITNs) as a major strategy for the control of malaria. Indoor residual spraying (IRS) of insecticides was used in 4,000 households in 2002 [10] but extended use of this technique, which has been effective in other regions of southern Africa, would also be limited by inadequate knowledge of vector species distribution and the lack of skilled staff.

During April 2001, the senior author (NC) visited Angola at the request of WHO, to assist in "strengthening malaria vector control especially the promotion of insecticide-treated bed nets". As one part of this mission, a rapid mosquito survey was carried in some of the malarious regions of the country with a focus on areas around the capital Luanda, the semi arid coastal cities of Benguela and Lobito and Huambo city in the tropical highlands. The main malaria transmission season in Angola lasts from November to April. This paper is based on data from that rapid survey and subsequent laboratory studies. The primary objective has been to gather information on species composition within the *An. gambiae *and *An. funestus *complexes, their potential role in malaria transmission and to provide information on the status of *kdr*-based resistance to pyrethroids in *An. gambiae *in Angola.

### Mosquito collections

**Figure 1:**
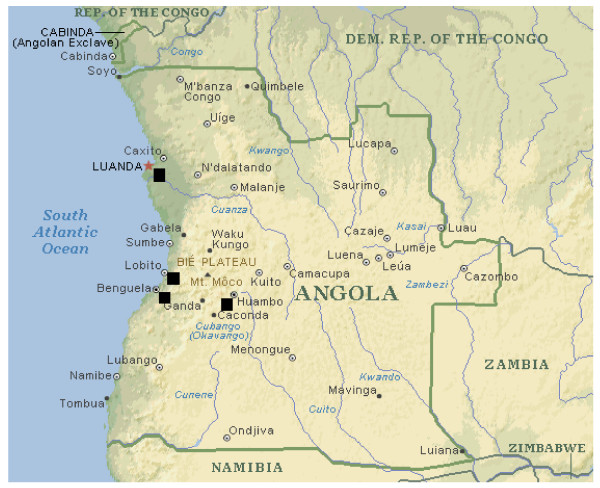
Map showing the localities in Angola where anopheline mosquitoes were collected: Luanda (Samba and Cacuaco), Lobito (São Pedro), Benguela (Cawanga and Bela Vista) and Huambo (São Jose and Cazenga).

Mosquitoes were collected towards the end of the rainy season during the period 11–19 April 2001 from seven localities in Angola, all close to or within urban areas; five in the semi-arid, coastal strip south from Luanda to Benguela and two in the humid tropical highlands of Huambo Province, (see Figure 1). viz: Funda in the coastal municipality of Cacuaco (08°51' S; 13° 34' E) just northeast of Luanda; Samba, a municipality of Luanda (08° 59' S; 13° 15' E); São Pedro, in the municipality of Lobito (12° 20' S; 13° 34' E); Cawango and Bela Vista, in the municipality of Benguela (12° 34' S; 13° 24' E); São Jose and Cazenga, in the city of Huambo (12° 47' S; 15° 44' E).

Indoor resting mosquitoes were collected from houses using pyrethrum spray collections and stored dry over silica gel in Eppendorf tubes. In Samba, only larvae were collected; these were obtained from a rainwater pool and preserved in 80% alcohol.

### Identification of species, molecular forms and *kdr *mutations

DNA was extracted from individual mosquitoes [11] and members of the *An. gambiae *complex were identified to the species-level and molecular-form using the protocol of Fanello *et al*. [12]. Identification of specimens of *An. funestus s.l*. was performed with the primers and protocol of Koekemoer *et al*. [13]. All identifications of M and S forms were conducted twice (by NC and KSC).

The presence of *kdr *alleles conferring knock-down resistance in West Africa was assessed using the primers and protocol of Martínez-Torres *et al*. [14]. The PCR products of samples collected in the field in Angola were run on agarose gels with control samples known to represent homozygous resistant (kdr/kdr), heterozygous (kdr/+) and homozygous susceptible (+/+) individuals. The PCR amplification products consisted of an internal control of 293 bp present in all specimens, a susceptible allele product of 137 bp and a resistance allele (*kdr*) band of 195 bp. To eliminate errors in scoring, all *kdr *allele assays were carried out twice (by NC and KSC) and discrepancies checked.

Sporozoites rates for *Plasmodium falciparum *were determined by the ELISA method [15,16], using the head and thorax of individual female mosquitoes.

## Results

The numbers of mosquitoes found resting indoors at each locality in Angola are shown in Table 1; data for *Culex *spp are included for comparative purposes but are not discussed further here. The numbers of individuals for the different species and/or molecular forms of the *An. gambiae *complex are shown in Table 2. Of the *An. gambiae s.s*. collected, the M-form was predominate, representing 93.5% (n = 403). The S-form was restricted to localities in the municipality of Benguela on the coast, where it comprised 3.8% (n = 184), and Huambo in the humid highlands, where it comprised 20% (n = 94) of *An. gambiae *s.s. collected. These differences in proportions of S-forms between coastal and highland sites are significant (p < 0.001). No M/S heterozygotes were found.

**Table 1:** Numbers and densities of adult mosquitoes of *An. gambiae *s.l., *An. funestus s.l*. and *Culex *from indoor pyrethrum spray collections.

			*An. gambiae s.l*.		*An. funestus s.l*		*Culex sp*.	
Muncipality	site	no. of houses	no.	no. per house	no.	no. per house	no.	no. per house
Benguela	Bela Vista	24	124	5.2	0	-	0	-
	Cawango	33	254	7.7	0	-	133	4.0
Cacuaco	Funda	29	29	1.0	315	10.9	0	-
Lobito	São Pedro	35	824	23.5	0	-	69	2.0
Huambo	Cazenga	34	31	0.9	0	-	105	3.1
	São Jose	37	121	3.3	0	-	313	8.5

**Table 2:** Numbers of M and S molecular forms of *An. gambiae *collected in Angola. Data are for indoor pyrethrum spray collections, except where noted.

Locality	Site	Molecular form		Totals
		M	S	
Benguela	Cawango	115	3	118
	Bela Vista	62	4	66
Lobito	São Pedro	91	0	91
Cacuaco	Funda*	13	0	13
Luanda	Samba**	21	0	21
Huambo	São Jose	57	7	64
	Cazengue	18	12	30
Totals		377	26	403

* 26 of 29 specimens successfully amplified; 13 of these were *An. melas*

** 22 identified as larvae; one of these was *An. arabiensis*

*An. melas *was found, together with *An. gambiae *M-form, resting indoors in the coastal area of Cacuaco. In the larval collection from Samba, one *An*. *arabiensis *was found along with 21 specimens of *An. gambiae *M-form. Forty-six specimens of *An. funestus s.l*. from indoor collections in Cacuaco were identified to species; all proved to be *An. funestus s.s*.

The results of assays for kdr resistance alleles in *An. gambiae *M and S-forms are shown in Table 3. All 218 individuals examined were exclusively of the homozygous susceptible genotype.

**Table 3:** Scoring of kdr genotypes *in An. gambiae *M and S-forms. All were homozygous susceptible +/+

Muncipality	site	*An. gambiae*		+/+
		M	S	
Benguela	Cawango	37	3	40
	Bela Vista	80	3	83
Lobito	São Pedro	41	0	41
Huambo	São Jose	27	7	34
	Cazenga	10	10	20
	Total	195	23	218

Footnote: kdr genotypes were not scored for the 13 specimens from Cacuaco

The results of sporozoite ELISA assays are shown in Table 4. Adult mosquitoes collected from Huambo had to be preserved in alcohol and hence were unsuitable for ELISA. The overall infection rate was 0.7 % in *An. funestus *(n = 140) and 1.9 % in *An. gambiae *(n = 580).

**Table 4:** *Plasmodium falciparum *sporozoite rates based on ELISA in mosquitoes of the *An. gambiae *complex and *An. funestus *collected in Angola

Municipality	Locality	*An. gambiae *% positive (n)	*An. melas *% positive (n)	*An. funestus *% positive (n)
Benguela	Bela Vista	0.8% (118)	-	-
	Cawango	1.5% (132)	-	-
Lobito	São Pedro	2.5% (317)	-	-
Cacuaco	Funda	0% (13)	0% (13)	0.7% (140)
Total		1.9% (580)	0% (13)	0.7% (140)

## Discussion

Little is known of malaria vectors in Angola and, with one exception (discussed below), all published studies preceded the war of independence and subsequent civil war, which finally ended in 2002. Although Angola is building up a cadre of technical staff to assist in the distribution of ITNs [10], as yet it has insufficient numbers of skilled malaria entomologists and data on malaria vectors remains scarce. Thus, despite the limitations of a rapid, time-limited survey, the data shown are a useful guide to the further studies needed for effective implementation of vector control.

A recent analysis of published and unpublished data on the distribution of the molecular forms of *An. gambiae *[6,17] has demonstrated how the M-form shows the greatest latitudinal range in West Africa, being the only form recorded in the Sudan and Sahel savannah areas of northern Senegal. The predominant domiciliary malaria vector in our study was *An. gambiae *M-form. The low proportion of the S-form among *An. gambiae *in Benguela and its absence in samples from Lobito and Luanda mirrors the findings in Carrara *et al*. [17] and almost certainly reflects the semi-arid climate of the coastal provinces. The more humid tropical highlands around Huambo would favour the greater proportion of S-form (20%) found there. In the published abstract of Carrara *et al*. [17] data are given on the distribution of M and S-forms from sites in Angola. For samples from around Luanda and Namibe, the M-form was overwhelmingly predominant. Namibe is close to the northern extension of the Namib desert. Sample sizes from inland sites in their study were very small (≤ 18) but showed a preponderance of S-form.

In our study we found a sporozoite rate in *An. gambiae s.s*. of 1.9% (95% CI 0.8–3.0), which is based on samples from localities where the M-form was predominant (97.6%). Despite these samples being collected in the rainy season when nullipars predominate, it is nevertheless higher than the 0.4% for *An. gambiae s.s*. in the studies of Carrara *et al*. [17].

Only a single *An. arabiensis *specimen was found in this study, in a larval collection. The scarcity of this species in samples from a semi-arid region that might have been expected to favour this species may be a reflection of seasonal abundance or exophily, although it is notable that this species was also scarce in the studies of Carrara *et al*. [17].

There is evidence (reviewed in [6]) that throughout its distribution, the M-form may more often be found in semi-permanent and man-made breeding sites. This suggests that consideration should be given to suitable environmental modifications to reduce mosquito breeding in the coastal peri-urban centres of Angola where M-form predominates. Environmental management would form a useful adjunct to ITNs and the residual spraying of houses [18]. Where S-form is more common, as in the highlands around Huambo (this study) or in the humid tropics of the Cabinda enclave [17], such measures could be less effective, since in many parts of its range, S-form readily breeds in small rain-dependent sites that are not as amenable to environmental management. In West Africa, there is evidence of varying levels of hybridisation between M and S-forms, a mechanism by which adaptive genes may flow from one to the other, including those conferring insecticide resistance [7,9]. In our study, no hybrids have been found out of 403 specimens that were typed. This infers a frequency of hybridisation of less than 1%, although in the *An. gambiae *complex, hybridisation between species and forms may be more likely at certain seasons as densities are undergoing change.

In this study we tested only 218 *An. gambiae s.s*. mosquitoes for kdr resistance and no resistance alleles were found. We did not expect to find resistance to pyrethroids, since these insecticides have not been available in significant quantities during the periods of civil strife, when agricultural production was severely disrupted. Carrara et al. [17] reported "the presence of the *kdr*-allele (about 18%)" in the Cabinda enclave, but did not find the resistant alleles in samples (of unstated size) from other sites in Angola. Whilst kdr resistance does not yet appear to have a highly significant effect on the performance of ITNs for malaria control, it would be prudent to increase monitoring for resistance using standard WHO bioassays and molecular tests that can detect both metabolic resistance and target site insensitivity [19,20].

## Authors' contributions

NC carried out the field collections of malaria vectors in Angola together with staff of the National Malaria Control Programme. He conducted the laboratory analysis of sporozoite rates and the PCR assays for identification of the *An. funestus *complex.

NC and KSC jointly carried out PCR assays for species and molecular forms of the *An. gambiae *complex, and the assays for *kdr*. All assays of molecular forms and kdr resistance were double-checked and any discrepancies resolved. HT advised on the experimental methods, results and interpretation of these assays.

NC and HT took the lead role in data analysis and interpretation. All three authors were closely involved in writing the paper and read and approved the final manuscript.
